# Liquid-Phase Chemical Melting Deposition for Anchored Nanoparticle–Nanofiber Architectures

**DOI:** 10.1021/acsanm.6c01148

**Published:** 2026-05-03

**Authors:** Hiep Pham, Kiernan O’Boyle, Gracie Boyer, Jonghyun Park

**Affiliations:** † 14717 Department of Mechanical and Aerospace Engineering Missouri University of Science and Technology, Rolla, Missouri 65409, United States; ‡ Department of Electrical and Computer Engineering Missouri University of Science and Technology, Rolla, Missouri 65409, United States

**Keywords:** chemical melting deposition, dual-extrusion electrospinning, anchored nanoparticles, metal oxide/carbon composite, lithium-ion battery anodes

## Abstract

We report chemical melting deposition (CMD), a manufacturing strategy designed to overcome the low mass loading and weak interfacial bonding inherent to vapor-based synthesis. Unlike conventional vapor routes, CMD leverages a transient liquid-phase transfer (TLPT) mechanism driven by the differential thermal degradation of carrier fibers to transfer and anchor nanoparticles directly onto target fibers. This process thermodynamically drives the wetting and interfacial fusion of nanoparticles, establishing a liquid-phase contact pathway that enables markedly higher active material loading. To validate the structural resilience of this fused architecture against extreme volumetric stress, we utilized lead oxide (PbO) as a model system, which typically suffers from catastrophic volume expansion (∼233%) in lithium-ion batteries. Mechanistic studies reveal robust and tunable particle–fiber attachment governed by CMD parameters, enabling optimized structural stability and electrochemical performance. The resulting PbO–carbon nanofiber (CNF) composite anode features a strain-accommodating hierarchical architecture via a self-buffering matrix, delivering a specific capacity of 466.8 mAh·g^–1^ at 200 mA·g^–1^ (∼1.2C) over 250 cycles, nearly doubling that of bare CNFs (235.3 mAh·g^–1^). These findings establish CMD as a highly versatile, liquid-phase manufacturing platform with implications extending far beyond conventional energy storage systems. This broadly applicable route provides a versatile methodology for designing high-loading, structurally integrated nanocomposites for diverse mechanically demanding applications, including advanced catalysis, sensors, and energy storage.

## Graphical abstract

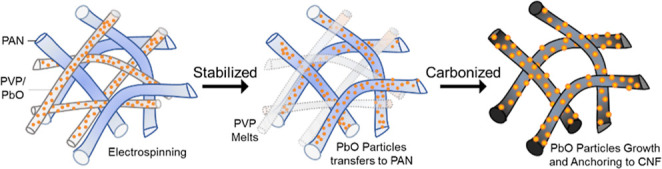

## 1 Introduction

Creating composite architectures where metal nanoparticles are robustly anchored onto nanostructured supports represents a “holy grail” challenge in materials engineering. Such architectures harness the synergies of each component, unlocking transformative properties for applications ranging from energy storage and conversion to high-performance filtration and catalysis.^1–3^ While embedding nanoparticles into carbon nanofibers (CNFs) has shown promise, their widespread translation into these multidisciplinary fields—from structural catalysts to flexible sensors—is bottlenecked by a universal grand challenge: achieving an exceptionally high mass loading of active nanoparticles without compromising the robust interfacial bonding required to withstand severe chemo-mechanical stresses.^4,5^

Conventionally, various methods have been utilized to fabricate these composites, yet each comes with significant trade-offs. For instance, metal particles were grown directly on 3D WO_3_ nanostructures through a solution-based synthesis involving the in situ redox reaction of WO_2.72_ nanowires to enhance the interaction between the metal particle and the nanostructured support.^6^ Similarly, gold and platinum nanoparticles were incorporated into tungsten oxide nanoneedles via aerosol-assisted chemical vapor deposition (CVD) for selective gas sensing.^7^ Another approach involved dispersing palladium nanoparticles onto and inside multiwalled carbon nanotubes through capillary filling with palladium precursor salts, followed by thermal treatment.^8^ However, these methods often suffer from low particle loadings, limited interface bonding, and low overall yield, which hinder their practical use in applications that require high active material contents. Additionally, vapor-phase techniques such as CVD are energy-intensive and time-consuming, often producing only microgram-scale composites after several hours of processing. Furthermore, the intrinsic “line-of-sight” limitation of CVD often precludes uniform deposition within deep porous structures due to shadowing effects, leaving internal surfaces uncoated.

In this study, we present a manufacturing strategy: chemical melting deposition (CMD). Unlike traditional approaches, CMD leverages the differential thermal degradation of a sacrificial polymer carrier to create a transient liquid-phase transfer (TLPT) mechanism. This transient liquid phase acts as a vehicle to transport and thermodynamically drive the wetting and interfacial fusion of metal precursors directly onto the target nanofiber scaffold during a single thermal treatment step. By facilitating intimate liquid-phase wetting before carbonization, CMD overcomes the weak adhesion typical of solid–solid interfaces, enabling the rapid fabrication of robust, high-yield composites with exceptional structural integrity. The CMD methodology leverages the differential thermal degradation of the polymer carriers—specifically, the melting of the sacrificial PVP carrier (∼150–180 °C) prior to the carbonization of the cross-linked PAN scaffold. Because the TLPT is driven by these polymer dynamics rather than the inherent chemistry of the active material, precursor particles dispersible within the carrier polymer can theoretically be transferred. By utilizing PbO as a model system with significant volumetric changes, we demonstrate that this mechanism creates a stable interfacial bond capable of accommodating substantial chemo-mechanical stress, offering a potential framework for evaluating other metal oxide/carbon systems. While existing electrospinning techniques, such as coaxial electrospinning,^9^ successfully encapsulate active materials within a core–shell structure, this geometry can sometimes restrict electrolyte accessibility to the core. Alternatively, the CMD strategy utilizes a side-by-side dual-extrusion approach designed to actively transport and anchor particles onto the outer surface of the structural scaffold. Furthermore, compared to traditional melt-infiltration^10^—which often requires separate postprocessing steps to infuse active materials into preformed porous carbons—CMD aims to achieve particle anchoring during a continuous thermal treatment step.

To validate the efficacy of this concept, lead­(II) oxide (PbO) was deliberately selected as a highly demanding model system to rigorously evaluate the mechanical resilience of the interface. Because PbO undergoes a catastrophic volumetric expansion (∼233%) upon lithiation,^11–13^ it typically obliterates conventional solid–solid physical interfaces. Successfully anchoring PbO using CMD provides strong structural evidence that our transient liquid-phase fusion can withstand the most severe chemo-mechanical degradation, thereby validating its universal applicability to any structural nanocomposite. While incorporating Pb with carbon can mitigate stress,^14–19^ previous approaches often required complex reaction conditions leading to low yields.^16,17,20,21^ For example, early studies on Pb/C composites prepared by solvothermal synthesis lacked mechanical robustness under long-term cycling.^14,19^ More advanced nanostructured composites confining Pb nanoparticles often required complex conditions such as hydrogen atmospheres or delicate decanting steps, which limited scalability.^16,17^ Moreover, most efforts focused on carbon nanoparticles, neglecting the unique structural advantages of CNFs, which offer high surface area and superior mechanical strength.^22^ Therefore, PbO serves as an ideal candidate to demonstrate the efficacy of CMD: if our CMD-engineered interface can accommodate these severe volumetric strains, it offers a powerful solution for mechanically demanding composite system.

In the CMD method, PbO nanoparticles are anchored and fused onto polyacrylonitrile (PAN)-derived CNFs via a dual-extrusion electrospinning process (Figure 1), followed by a controlled thermal treatment. This process establishes a chemically fused interface superior to simple physical adhesion, allowing the CNF matrix to effectively buffer the volume expansion of PbO. Through this approach, we demonstrate that the CMD-derived PbO–CNF (PCNF) composite delivers superior electrochemical performance and structural stability compared to bare CNF, offering a generalizable blueprint for the design of next-generation multifunctional nanomaterials.

**Figure 1:**
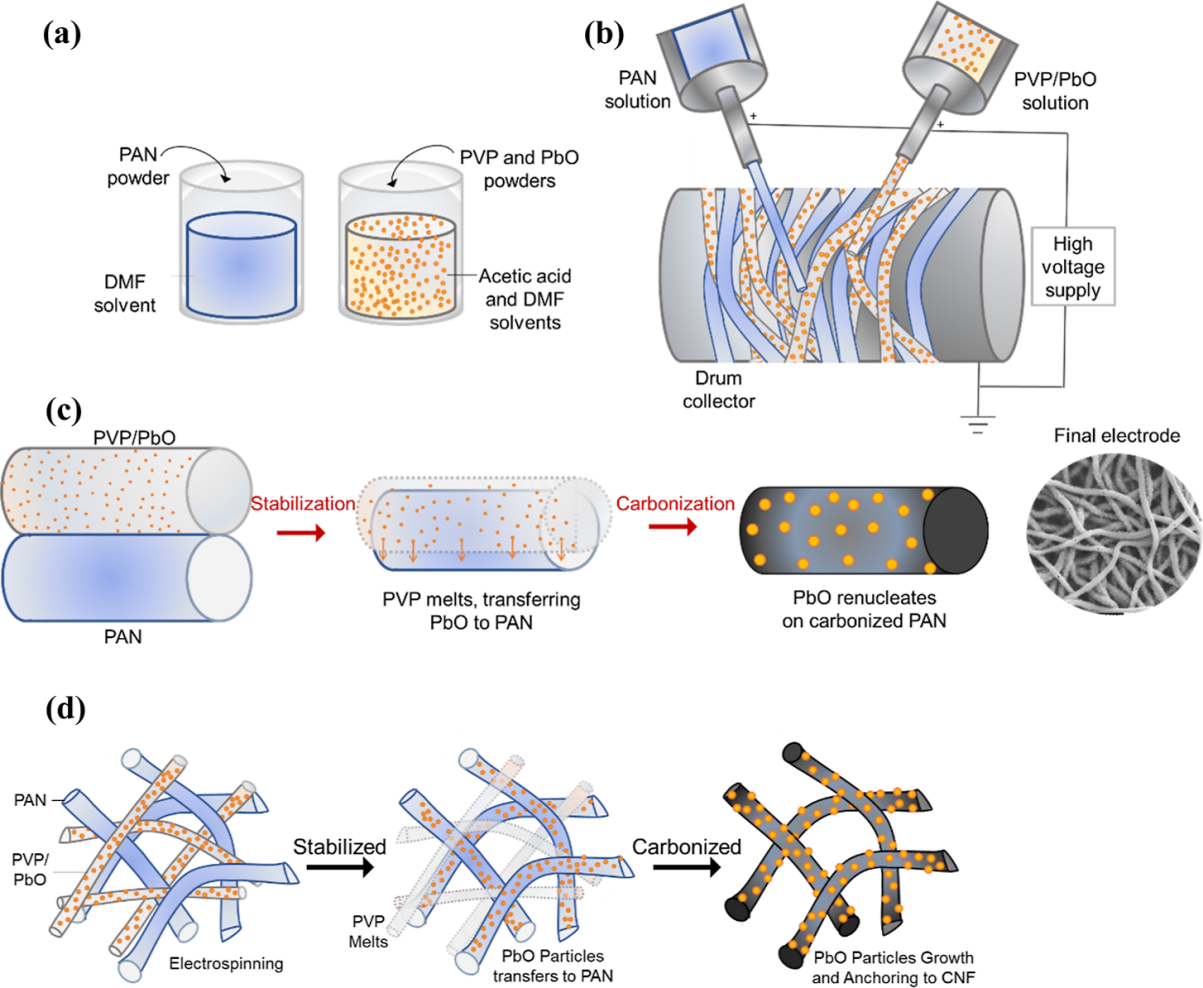
Schematic illustration of the chemical melting deposition (CMD) process. (a) Preparation of chemically incompatible precursor solutions: structural PAN in DMF and sacrificial PVP/PbO in DMF/acetic acid. (b) Dual-extrusion electrospinning setup enabling the simultaneous deposition of carrier and structural fibers. (c) The fundamental CMD mechanism: thermal treatment induces the melting of PVP, creating a transient liquid phase that transfers PbO nanoparticles onto the PAN scaffold before carbonization. (d) Structural evolution of the PCNF network, highlighting the thermodynamically driven wetting and anchoring of active materials onto the carbon nanofiber scaffold.

## 2 Experimental Section

### 2.1 Fabrication of PbO–CNF Anodes via Chemical Melting Deposition

The PbO solution was prepared by dispersing 1.5 g of PbO powder in 5 mL of acetic acid using a SpeedMixer (FlackTek) for 10 min until complete dissolution. A second solution was obtained by dissolving 1.0 g of PVP in 5 mL of DMF, and the two solutions were subsequently combined and stirred for 2 h to form a homogeneous PbO–PVP mixture. A separate structural solution was prepared by dissolving 1.2 g of PAN in 10 mL of DMF, and stirred at 60 °C for 4 h. These two syringes—one containing the PbO–PVP solution and the other the PAN solution—were mounted on opposite sides of a rotating drum collector wrapped with aluminum foil (Figure 1). A voltage was applied to the syringe tips, and electrospinning was performed at an ambient temperature of 50 °C. The effects of electrospinning parameters, including applied voltage and extrusion rate, were systematically investigated. After deposition, the fibers were peeled from the aluminum foil and air-dried for 12 h. The dried fibers were then subjected to calcination. They were first stabilized at 270 °C for 3 h (ramp rate: 5 °C min^–1^) and then carbonized at 800 °C for 1 h under nitrogen with a heating rate of 2 °C min^–1^. After cooling to room temperature, the resulting PCNF mats were collected, punched into 16 mm disks, and transferred into an argon-filled glovebox for cell assembly. Pure CNFs were also prepared using the same conditions without PbO addition for comparison. The electrochemically active material loading was approximately 4–6 mg per electrode for PCNF samples and ∼3 mg per electrode for pure CNFs. This electrochemically active mass corresponds to an areal loading of ∼2.0 to 3.0 mg cm^–2^ for the PCNF composite electrodes. The morphologies of PCNF and CNF samples were characterized by field-emission scanning electron microscopy (FE-SEM, FEI Helios 600 Nanolab) equipped with energy-dispersive X-ray spectroscopy (EDS) and by transmission electron microscopy (TEM, Tecnai F20). X-ray photoelectron spectroscopy (XPS) was performed to confirm the chemical composition and bonding states. Thermogravimetric analysis (TGA, TA Instruments) was conducted to quantify PbO loading, where samples (∼300 mg) were heated to 600 °C at a rate of 5 °C min^–1^ under nitrogen.

### 2.2 Electrochemical Measurements

CR2032-type coin cells were assembled in an argon-filled glovebox for electrochemical analysis. Lithium metal foils (0.75 mm thick, Sigma-Aldrich) served as counter and reference electrodes. Celgard 2325 membranes were used as separators, and a 1 M LiPF_6_ solution in ethylene carbonate (EC) and dimethyl carbonate (DMC) (1:1 v/v) was used as the electrolyte. Galvanostatic charge–discharge cycling was performed in the voltage range of 0.01–3.0 V. Long-term cycling stability was evaluated by applying 100 mA g^–1^ for 15 formation cycles, followed by 200 mA g^–1^ for 250 cycles. Rate performance (C-rate) tests were conducted sequentially at 100, 200, 500, and 1000 mA g^–1^, followed by a return to 100 mA g^–1^, with five cycles per step. All galvanostatic tests were performed using a NEWARE battery testing system. Cyclic voltammetry (CV) and electrochemical impedance spectroscopy (EIS) measurements were performed using an IVIUMnSTAT potentiostat (Ivium Technologies). The CV scans were conducted in the potential range of 0.01–3.0 V at a scan rate of 0.25 mV s^–1^, and the EIS measurements were carried out over the frequency range of 1 MHz to 10 mHz with a 10 mV amplitude.

## 3 Results and Discussion

### 3.1 CMD Fabrication Principle

To overcome the inherent limitations of conventional synthesis methods—such as poor mass loading and weak physical adhesion—the CMD process was designed to facilitate direct liquid-phase integration. This approach represents a notable alternative from solid-state mixing to thermodynamically driven liquid wetting. Unlike traditional methods that rely on the physical contact of solid particles, CMD exploits the differential thermal behavior of two immiscible polymer precursors. By utilizing a sacrificial carrier that melts and flows before the structural scaffold carbonizes, we induce a transient liquid flux that actively transports and embeds nanoparticles onto the target surface. Figure 1 systematically details the overall architecture and manufacturing principles of this process. Figure 1a illustrates the preparation of the chemically incompatible precursor solutions. The experimental configuration involves a dual-extrusion electrospinning system (Figure 1b), where two distinct precursor jets—one carrying the active material in a sacrificial polymer (PVP/PbO) and the other serving as the structural scaffold (PAN)—are simultaneously deposited onto a rotating collector. The fundamental mechanism of particle anchoring is visually summarized in Figure 1c,d. During the initial stages of thermal treatment, the sacrificial PVP fiber reaches its glass transition and melting points (∼150–180 °C) while the PAN fiber remains structurally intact. This differential thermal behavior creates a transient liquid phase that thermodynamically wets the adjacent PAN fibers (Figure 1d), effectively transferring and fusing the PbO particles onto the scaffold surface before the final carbonization step.

### 3.2 Structural Evolution and Interpenetrating Networks

The formation of a robust, stress-dissipating architecture is governed by the synergistic relationship between the initial precursor assembly and its subsequent thermal transformation. In this section, the structural evolution of the PCNF composite is systematically dissected through its two defining fabrication stages: dual-extrusion electrospinning and controlled thermal treatment. We first establish how electrospinning parameters dictate the initial fiber morphology and entanglement, which serves as the physical template. Subsequently, the thermodynamic role of the heating profile is elucidated in activating the TLPT mechanism. Finally, the resulting interpenetrating nanofiber network is characterized, demonstrating how these optimized processing conditions yield a self-buffering matrix capable of sustaining high active material loading.

### 3.3 Electrospinning Optimization

The morphology of the resulting fibers is heavily dictated by the initial electrospinning parameters. Based on a preliminary screening of the Taylor cone stability window, multiple combinations of voltage and flow rates were investigated to determine the optimal conditions for particle transfer. From this broader set, two representative regimes were selected for detailed analysis to contrast the effects of polymer relaxation dynamics: a high-throughput/high-voltage condition (PCNF–H: 25 kV, 1 mL h^–1^) and a controlled low-throughput/low-voltage condition (PCNF-L: 15 kV, 0.5 mL h^–1^). Rather than isolating individual process variables, these paired conditions were selected to establish two distinct operational boundaries: a high-throughput, highly entangled regime (PCNF–H) and a slow-extrusion, relaxed fiber regime (PCNF-L). Evaluating the composite across these varying states of macroscopic entanglement provides insight into how precursor morphology influences the subsequent liquid-phase transfer process. The morphological differences between the as-spun fibers containing only PbO and the dual-spun composite fibers are compared in Figure 2. As shown in Figure 2a, the PbO was clearly present on the surfaces of the PVP nanofibers, indicated by numerous white spots anchoring the particles. Upon dual-spinning with the PAN solution (Figure 2b), the PbO-containing PVP nanofibers became less distinct due to the higher fiber yield of PAN; however, particles remained visible on select fibers, confirming their successful incorporation. This heterogeneous appearance is an expected characteristic of the simultaneous dual-extrusion process, which produces a mixed network of distinct pure PAN fibers and particle-bearing PVP fibers in the as-spun state. Evidence of global particle incorporation across the structural network is subsequently confirmed post-thermal treatment through TGA (Figure 5g) and corresponding SEM analysis. Figure 2c,d contrast the structural evolution before thermal treatment. PCNF–H exhibited a high degree of entanglement and junction formation (Figure 2c), whereas PCNF-L displayed long, continuous, and discrete nanofibers (Figure 2d). Notably, the nanofiber diameter of the PCNF-L sample was significantly larger than that of PCNF–H, a difference attributed to the slower extrusion rate and lower voltage providing sufficient polymer chain relaxation time. Post-thermal treatment analysis (Figure 2e) further revealed that during stabilization, the wrapped PVP melts around the PAN fibers—which retain their structure above 300 °C —effectively transferring the PbO to the PAN to facilitate robust particle-fiber bonding and junction formation. The proposed TLPT mechanism is corroborated by the distinct thermal-mechanical phase transitions of the selected polymers. Specifically, thermal analysis studies^23,24^ have established that PVP undergoes a prominent glass transition and subsequent melting flow within the 150–180 °C regime. In contrast, PAN is well-documented to undergo structural cyclization and thermal stabilization without passing through a liquid melt phase.^25^ The intermediate stabilization phase captured in Figure 2e provides direct morphological support for this transient state, illustrating the molten PVP actively wetting the structurally intact PAN scaffold prior to its subsequent volatilization.

**Figure 2:**
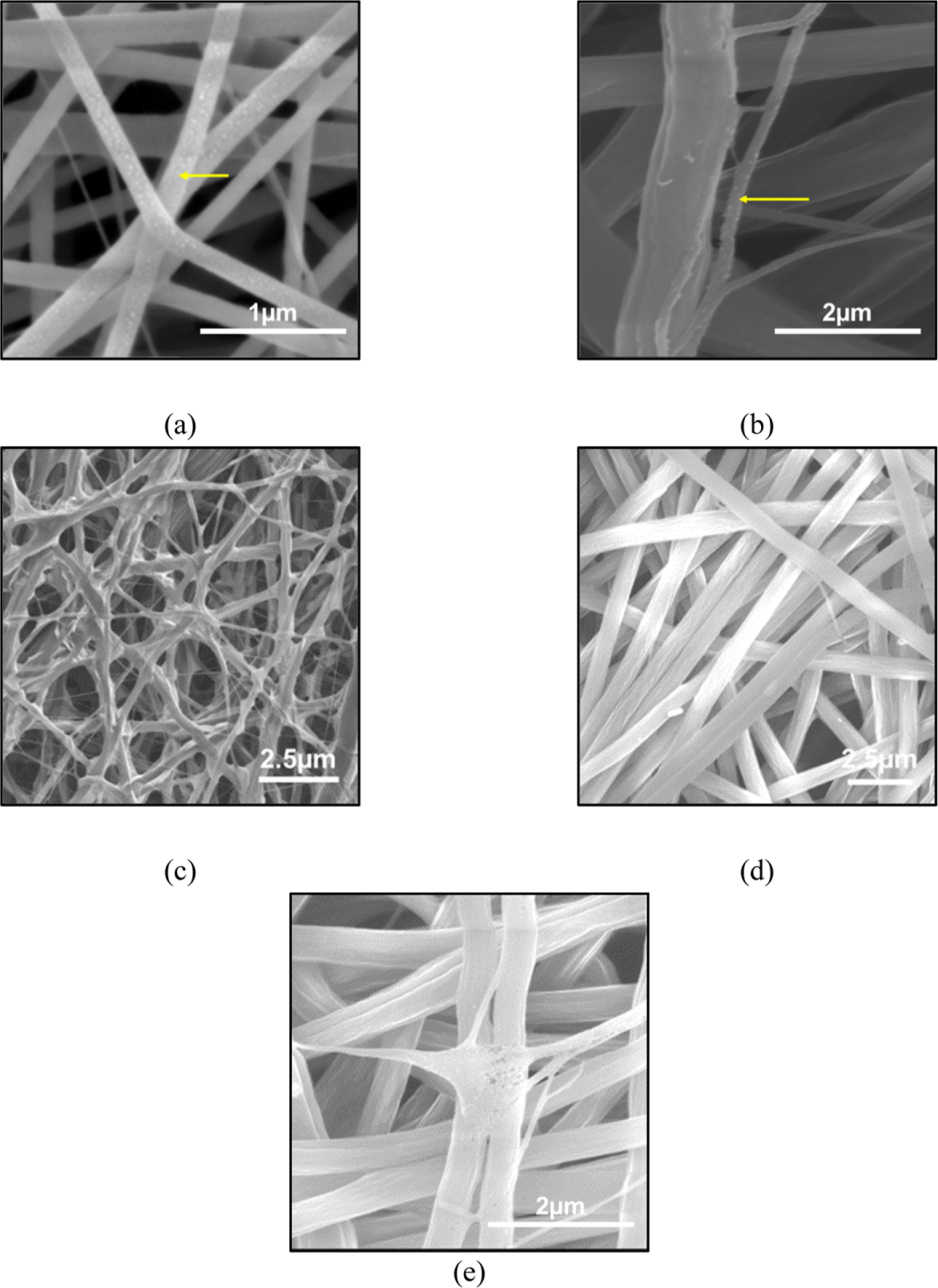
Morphological evolution of precursor fibers during the CMD process. SEM images of (a) as-spun sacrificial PVP/PbO nanofibers showing dense particle loading and (b) the dual-spun composite mat containing both PVP/PbO and PAN fibers. As-spun morphologies of (c) PCNF–H (25 kV, 1 mL h^–1^) showing a high degree of entanglement and (d) PCNF-L (15 kV, 0.5 mL h^–1^) exhibiting distinct, larger nanofibers due to optimized relaxation times. (e) Stabilized intermediate state showing the onset of the TLPT mechanism, where molten PVP wets the PAN surface, facilitating particle anchoring and interfiber junction formation.

### 3.4 Thermal Treatment Optimization

The thermal conversion profile, specifically carbonization temperature and heating ramp rate, is critical for defining the final electrode morphology and electrochemical performance. To determine the thermodynamic sweet spot for carbonization, the investigation was expanded across a temperature range of 600–900 °C using the PCNF–H parameters as a baseline. The electrochemical evaluation of these samples, presented in Figure 3, reveals a clear dependency on calcination temperature. The lowest capacities were observed for samples treated at 600 and 700 °C (Figure 3a,b), attributed to incomplete graphitization and limited conversion of the PAN precursor into conductive CNFs. While PVP carbonizes at these temperatures, the limited conversion of PAN results in higher internal resistance and incomplete structural contraction (Figure 3c). Conversely, increasing the temperature to 900 °C caused performance degradation (Figure 3a), likely due to the partial volatilization of the CNF scaffold and subsequent loss of anchored PbO particles. This degradation is likely influenced by carbothermic reduction (e.g., PbO + C → Pb + CO/CO), a reaction common when metal oxides are in intimate contact with carbon matrices at 900 °C. The consumption of the anchoring carbon scaffold and the lower stability of the resulting metallic lead at high temperatures can lead to structural isolation and active material loss. Consequently, 800 °C was identified as the optimal equilibrium. Furthermore, the heating ramp rate proved to be a decisive factor governing the TLPT mechanism. Figure 3d–f demonstrate that a slow ramp rate of 1 °C min^–1^ resulted in particle detachment (Figure 3d), whereas increasing the rate to 5 °C min^–1^ precipitated numerous larger PbO particles (Figure 3f). This confirms that a faster ramp rate sustains the transient liquid flux necessary for deep anchoring, as corroborated by TEM imaging (Figure 3g), which shows chemically fused interfaces between the PbO nanoparticles and the CNF scaffold. The localized TEM analysis (Figure 3g), observed in conjunction with the distinct electrochemical voltage plateaus that match established PbO conversion reactions (Figure 7a), supports the presence and preservation of the active PbO phase within the structural matrix following thermal treatment.

**Figure 3:**
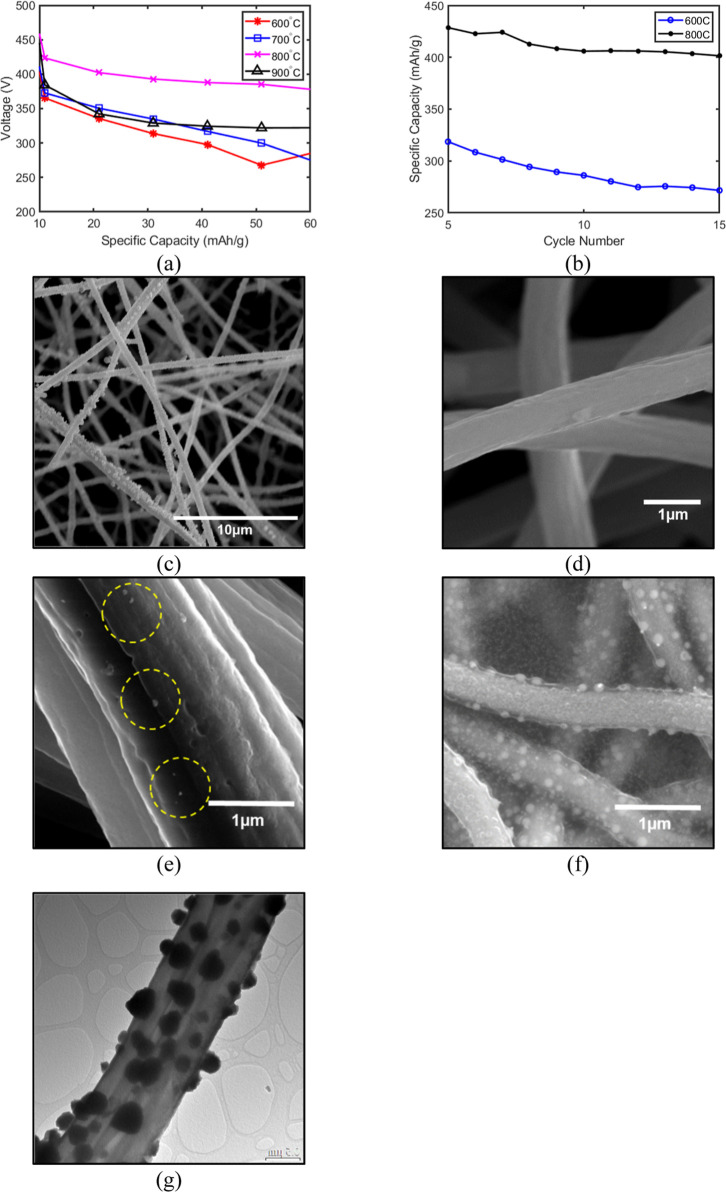
Optimization of thermal conversion parameters for enhanced structural integrity. (a) Voltage profiles of samples calcined at temperatures ranging from 600 to 900 °C. (b) Electrochemical capacity comparison between samples carbonized at 600 and 800 °C after five formation cycles, illustrating the necessity of complete graphitization. (c) SEM of the 600 °C sample showing incomplete carbonization. The critical effect of heating ramp rate on particle anchoring is shown in SEM images of samples treated at (d) 1 °C min^–1^ (particle detachment), (e) 2 °C min^–1^ (partial anchoring), and (f) 5 °C min^–1^ (robust anchoring via sustained liquid flux). (g) TEM micrograph of the 5 °C min^–1^ sample confirming the chemically fused, intimate interface between PbO nanoparticles and the CNF scaffold.

### 3.5 Sacrificial Carrier Validation

To further validate the sacrificial nature of the PVP carrier, the carbonization yields of isolated PVP and PAN fiber mats were compared (Figure 4). After identical thermal treatment at 800 °C, the PAN mat (Figure 4b, d) retained its integrity as a continuous CNF network. In stark contrast, the PVP mat (Figure 4a, c) underwent near-complete volatilization, leaving only fragmented residues. It should be noted that PAN mats commonly undergo macroscopic dimensional shrinkage during the thermal dehydrogenation and carbonization process at 800 °C, which accounts for the altered macroscopic shape observed in Figure 4d. However, at the microstructural level, it carbonizes into a freestanding nanofiber network, sharply contrasting with the complete vaporization of the PVP mat. It is posited that during CMD, the PVP melts onto adjacent PAN fibers to completely cover them along with the carried PbO and then volatilizes during carbonization, leaving behind the PbO that it carried to interact with the PAN-based CNF. This aligns with reports that PVP burns out completely from nanofibers at temperatures around 550 °C,^26^ explaining why an appreciable CNF mat could not be formed from the PVP precursor alone.

**Figure 4:**
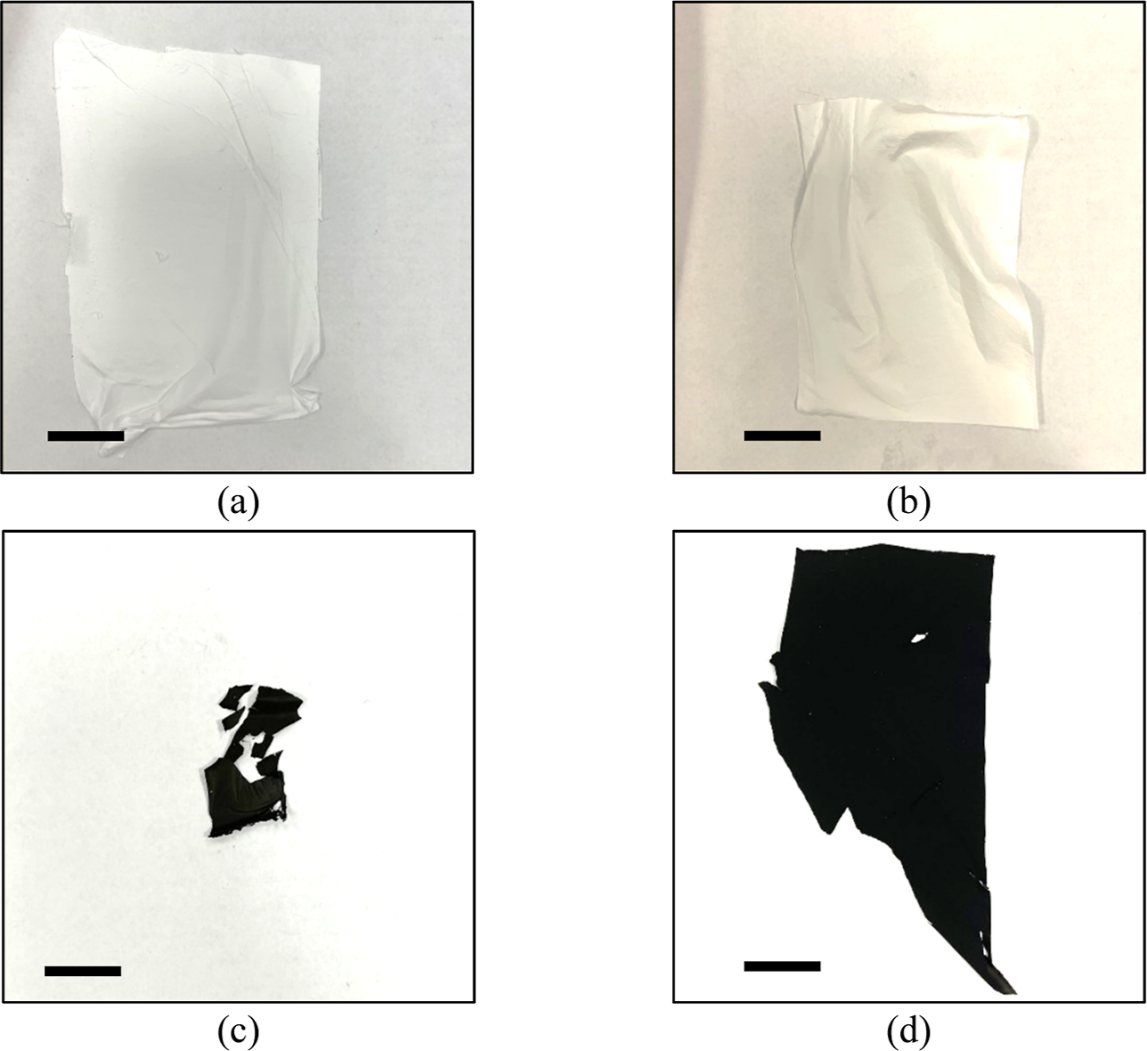
Validation of the sacrificial carrier mechanism. Digital photographs of (a) pure PVP and (b) pure PAN nanofiber mats before thermal treatment. Postcarbonization results show (c) the complete volatilization and fragmentation of the PVP mat, contrasting with (d) the structural integrity of the carbonized PAN mat. This confirms the role of PVP as a transient delivery vehicle. Scale bars: 1 in.

### 3.6 Interpenetrating Nanofiber Architecture

The optimized CMD process yields a hierarchical stress-dissipating structure, as illustrated in Figure 5. SEM imaging reveals that PCNF-L achieved more uniform particle dispersion compared to PCNF–H. The average fiber diameters were 799 nm for PCNF–H, 916 nm for PCNF-L, and 506 nm for pure CNF. PCNF–H exhibited a variation in thick and thin nanofibers, surmised to be mostly PAN and diminished PVP/PbO fibers, respectively. In contrast, PCNF-L displayed a more homogeneous dispersion (Figure 5d), with numerous particles showing strong attachment to the CNF surface. The larger fiber size in PCNF-L is explained by the electrospinning parameters which permitted a larger amount of PbO to be anchored. Importantly, both PCNF–H and pure CNF showed fiber entanglement with junctions where fibers appear fused together (Figure 5b, f). This interpenetrating nanofiber structure acts as a self-buffering matrix, bridging electron transfer along thinner nanofibers while using thicker fibers to provide mechanical support against volume expansion.^27^

**Figure 5:**
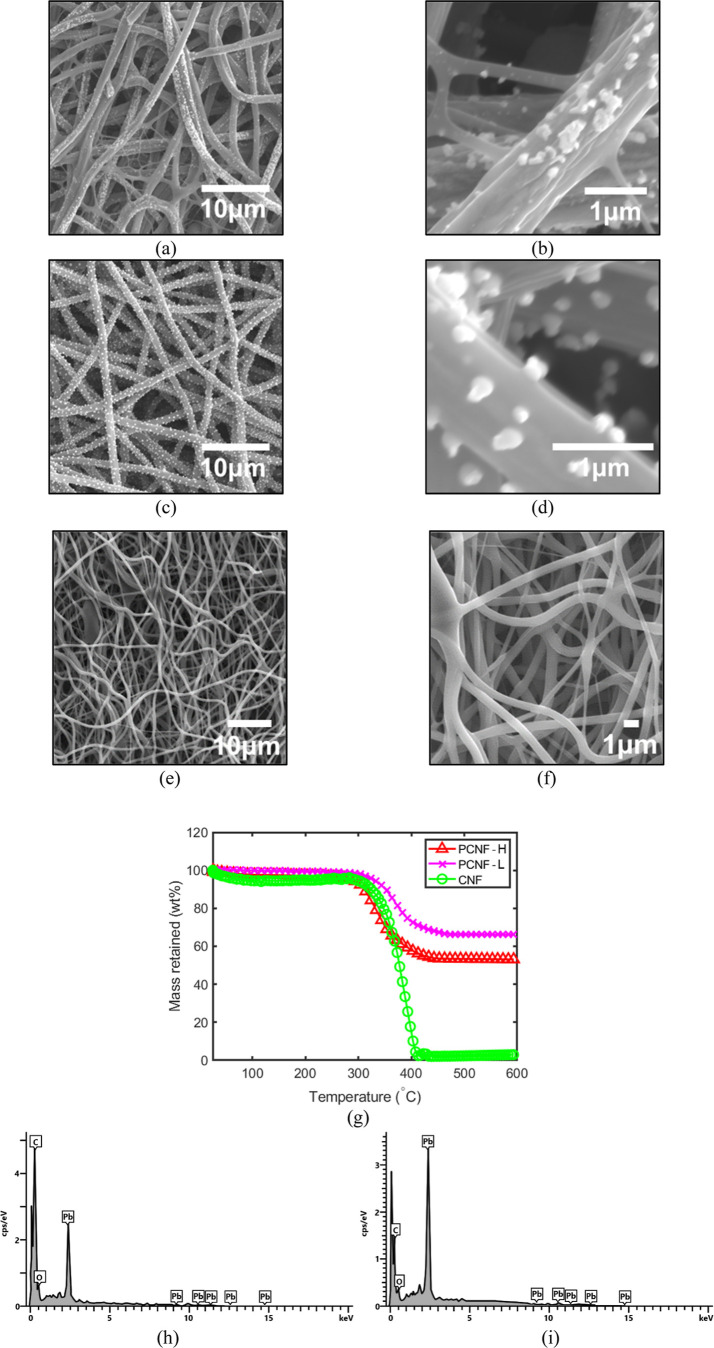
Microstructural characterization of the final CMD-engineered composites. SEM images at low and high magnification for (a,b) PCNF–H, showing a bimodal fiber distribution; (c,d) PCNF-L, displaying a uniform hierarchical network with higher loading PbO particles; and (e, f) pure CNF. (g) Thermogravimetric analysis (TGA) curves quantifying the high mass loading of PbO in PCNF–H (53.2%) and PCNF-L (66.1%), confirming efficient particle transfer. EDX-SEM elemental spectra for (g) PCNF–H and (h) PCNF-L.

A quantitative analysis of the PbO content was performed using TGA (Figure 5g). As the temperature increased to 300 °C, the curve of the TGA result remained nearly constant, until it reached 400 °C, at which the mass retained began to substantially decrease until the PCNF–H and PCNF-L stabilized after a 46.8 and 33.9% mass decrease, respectively. This indicated that the PbO content in the PCNF–H and PCNF-L could be as high as 53.2 and 66.1%. The starting PbO amount in the spinning solution for either sample was 41.7% considering the total amount of PAN, PVP, and PbO used. The enrichment of PbO content from the original amount after carbonization can be explained by the volatilization of carbon species from the PAN or PVP precursors, which increases the relative weight fraction of the nonvolatile PbO. The higher loading in PCNF-L is directly related to the more intimate wrapping between the starting precursor nanofibers, facilitating efficient liquid-phase transfer. Without this wrapping, the PVP carrying PbO evaporates without transferring PbO to the PAN precursor, causing particle loss. To further verify the elemental composition of the composites, EDX-SEM spectra were collected (Figure 5h,i). The spectra for both PCNF–H and PCNF-L exhibit prominent characteristic peaks for Pb, O, and C. This elemental confirmation aligns with the morphological observations, proving that the TLPT mechanism effectively anchors and retains the PbO active phase across the carbon nanofiber network throughout the high-temperature carbonization process.

### 3.7 Interfacial Chemical Characterization

To substantiate the “chemical fusion” driven by the TLPT mechanism beyond morphological inference, X-ray photoelectron spectroscopy (XPS) was conducted to systematically probe the interfacial bonding states and elemental compositions at different conversion stages (Figure 6). Within this study, “chemical fusion” refers specifically to the apparent formation of covalent linkages (such as the Pb–O–C environments suggested by XPS) across the interface, whereas “physical adhesion” is used to denote bonding that relies predominantly on weaker van der Waals interactions or geometric entrapment. A comparison between the sample calcined at 600 °C (incomplete CMD) and 800 °C (optimized CMD) reveals a distinct and profound transformation in the chemical nature of the nanoparticle-nanofiber interface.

**Figure 6:**
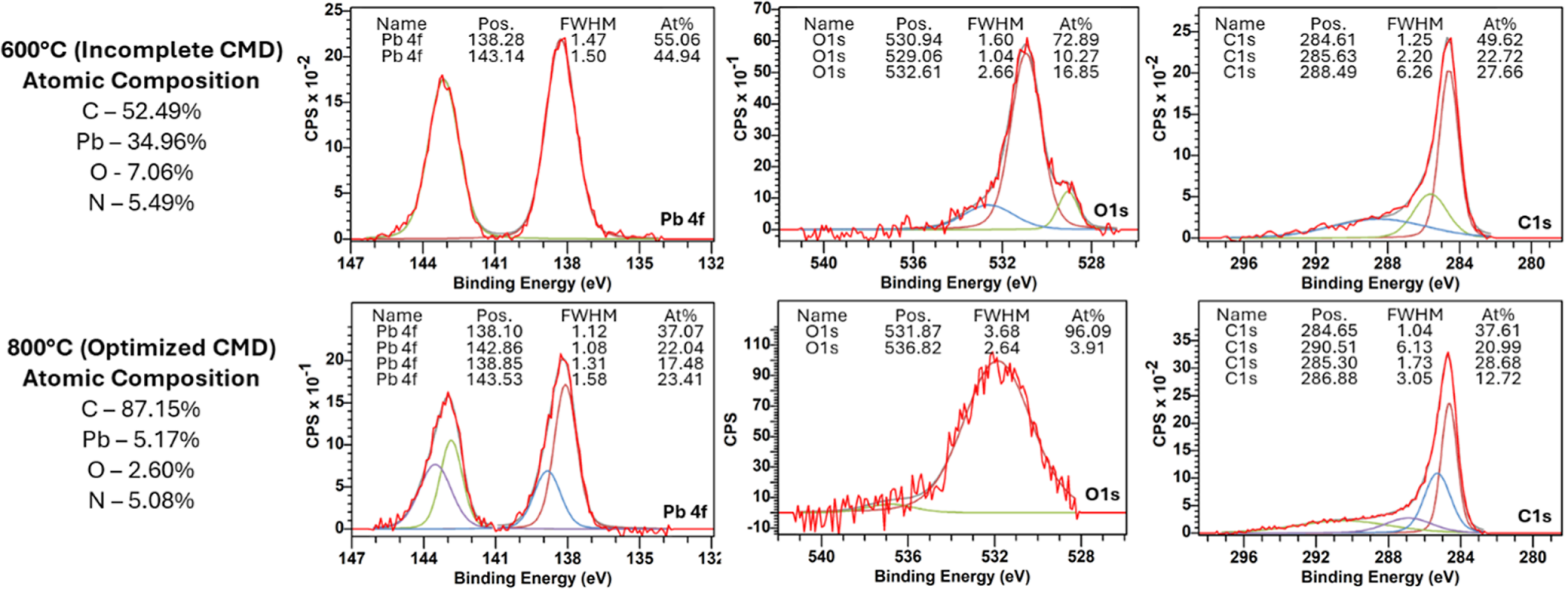
Interfacial chemical characterization via X-ray photoelectron spectroscopy (XPS). Comparative elemental composition and high-resolution core-level spectra (Pb 4f, C 1s, and O 1s) for the composite samples calcined at 600 °C (top) and 800 °C (bottom). The emergence of the high-binding-energy doublet in the Pb 4f spectra and the distinct shift to 531.87 eV in the O 1s spectra at 800 °C provide direct spectroscopic evidence for the formation of robust Pb–O–C covalent bonds, successfully validating the chemically fused nanoparticle-nanofiber architecture.

First, the overall atomic composition reflects the progression of the CMD process. At 600 °C, the carbon content is 52.49%, whereas at 800 °C, it significantly increases to 87.15%. This confirms the complete volatilization of the sacrificial PVP carrier and the successful graphitization of the PAN structural scaffold, setting the stage for robust interfacial anchoring. The most compelling evidence of chemical fusion lies in the high-resolution core-level spectra. In the Pb 4f spectra of the 600 °C sample, a standard, symmetrical doublet appears at 138.28 and 143.14 eV, which is indicative of the simple physical deposition of bulk PbO (Pb^2+^). Strikingly, upon successful CMD activation at 800 °C, the Pb 4f spectra deconvolute into two distinct pairs of doublets. Alongside the characteristic bulk PbO peaks (138.10 and 142.86 eV), a new, prominent high-binding-energy doublet emerges at 138.85 and 143.53 eV. This positive binding energy shift provides direct spectroscopic evidence of electron density withdrawal from the Pb atoms, strongly signifying the formation of Pb–O–C interfacial covalent bonds between the molten nanoparticles and the carbonizing matrix.

This chemical transition is fully corroborated by the O 1s spectra. The 600 °C sample exhibits multiple oxygen environments, including a peak at 529.06 eV corresponding to typical metal–oxygen (Pb–O) crystal lattice bonds. However, after the 800 °C treatment, this standard lattice oxygen peak virtually disappears, and the O 1s spectrum is entirely dominated by a single, massive peak shifted to 531.87 eV (accounting for 96.09% of the area). This specific binding energy is highly characteristic of oxygen species bridging a metal and a carbon lattice (C–O–Pb) and surface defect sites. Furthermore, while the broad O 1s envelope is dominated by the peak at 531.8 eV representing bridging oxygen and defect states, the asymmetric tail extending into the 529–530 eV range is attributed to the presence of residual lattice oxygen (Pb–O) from the preserved metal oxide phase. Furthermore, the C 1s spectra of the 800 °C sample show a dominant graphitic carbon peak (C–C/C=C) at 284.65 eV along with distinct oxygen-bound carbon peaks (C–O/C=O), confirming the integration of the newly formed Pb–O–C bonds into the robust carbon backbone. These high-binding energy shifts in both the metallic (Pb 4f) and oxygen (O 1s) core levels are established spectroscopic indicators often associated with interfacial coupling. As discussed in recent studies on metal-oxide/carbon interfaces,^28,29^ the withdrawal of electron density from the metal center toward a more electronegative oxygen–carbon bridge produces similar XPS signatures, strongly suggesting the formation of chemical linkages rather than relying solely on physical van der Waals adhesion. To address the potential contribution of ambient surface oxidation, it is noted that carbonization was strictly performed under a continuous inert nitrogen atmosphere up to 800 °C. Standard surface oxidation typically results in shifts predominantly toward bulk PbO or PbO_2_ states. While defect-induced shifts remain a factor, the observed high-binding-energy peak at 138.85 eV, coupled with the shift in the O 1s spectra toward bridging characteristics, aligns more closely with defect-anchored covalent integration rather than simple surface oxidation alone. Collectively, these comprehensive XPS findings directly validate that the transient liquid phase does not merely transport particles to the surface. Instead, it thermodynamically drives a deep interfacial chemical reaction, firmly and covalently anchoring the active nanoparticles into the nanofiber scaffold.

### 3.8 Electrochemical Performance

To ensure the reliability of the electrochemical evaluations, all presented charge–discharge profiles and cycling data reflect representative performances that were consistently verified across multiple identically prepared coin cells. The electrochemical stability of the CMD-engineered PCNF anodes was evaluated using coin-cell tests. Figure 7a presents the voltage profiles at a current density of 200 mA g^–1^. The initial discharge capacities were 454 and 661 mAh g^–1^ for PCNF–H and PCNF-L, respectively. Distinct voltage plateaus appear near 0.5 and 0.3 V, corresponding to the stepwise lithiation of PbO and subsequent alloying reaction.^30^ The reactions described by eqs 1 and 2 represent the processes governing the electrochemical behavior

1 $$PbO_{x}+2x{Li}^{+}+2xe^{-}\to Pb+x{Li}_{2}O,\left(x=1,2\right)$$

2 $$Pb+y{Li}^{+}+ye^{-}↔{Li}_{y}Pb,\left(0\leq y\leq 4.4\right)$$

**Figure 7:**
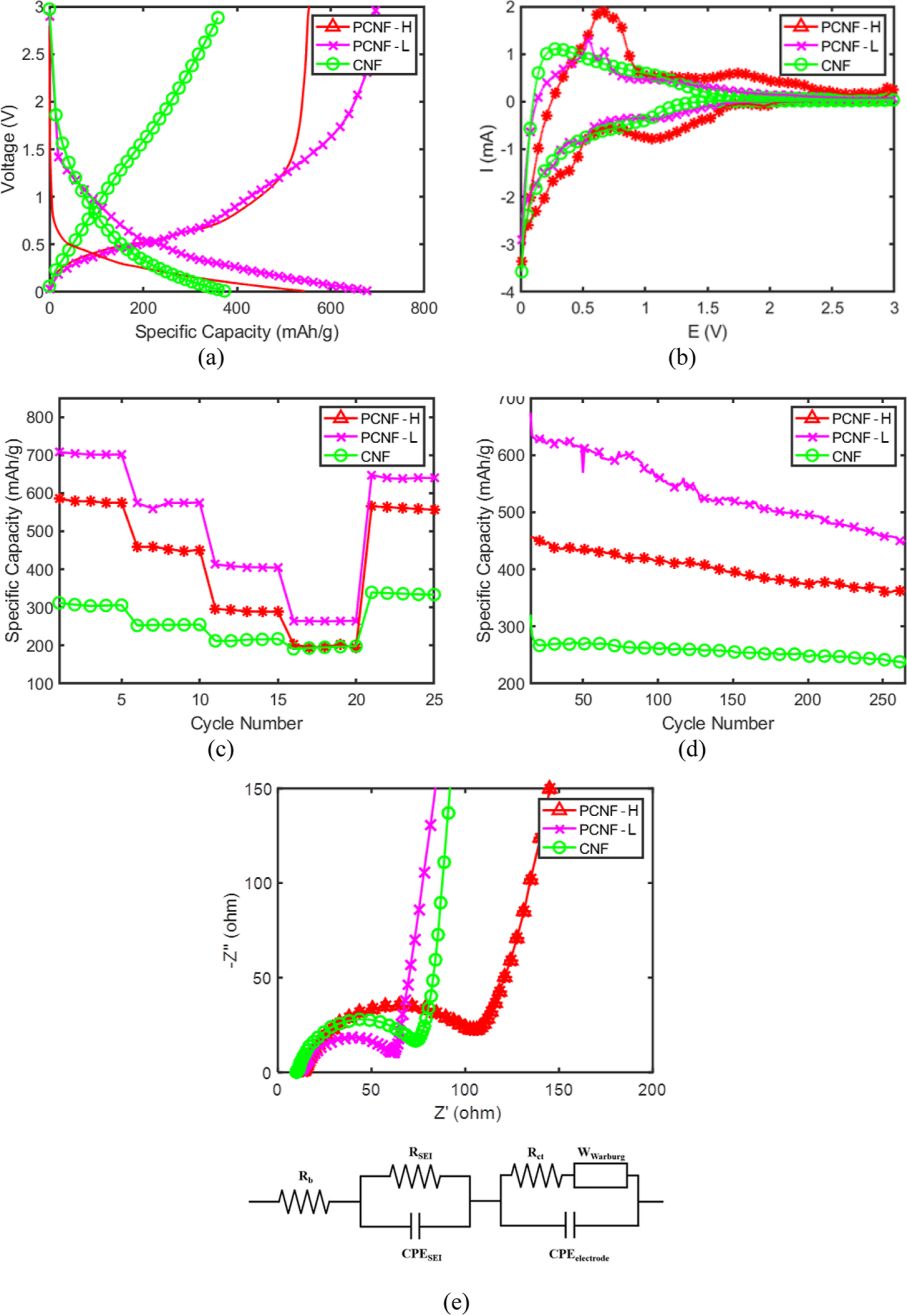
Electrochemical performance of CMD-engineered anodes. (a) Initial voltage profiles of PCNF samples at 200 mA g^–1^. (b) Cyclic voltammetry (CV) curves comparing the first cycle for all samples, showing reversible redox behavior. (c) Rate capability performance at current densities from 100 to 1000 mA g^–1^. (d) Long-term cycling stability at 200 mA g^–1^ over 250 cycles. (e) EIS Nyquist plots and equivalent circuit, where PCNF-L exhibits lower charge-transfer resistance (*R*_ct_), indicating enhanced interfacial kinetics.

A pronounced reduction peak between 0.5 and 0.9 V corresponds to the conversion of PbO to metallic Pb accompanied by the formation of Li_2_O (eq 1). At voltages below 0.5 V, intermediate Li_*x*_Pb alloys emerge (eq 2). During the initial formation cycles at 100 mA g^–1^, the electrodes experience an expected initial capacity loss. This phenomenon is a well-documented characteristic of conversion-alloying anodes, stemming from the irreversible formation of the solid–electrolyte interphase (SEI) and the extraction of oxygen to form amorphous Li_2_O matrices (eq 1). However, as shown in the postformation voltage profiles (Figure 7a, evaluated at 200 mA g^–1^), the Coulombic efficiency rapidly recovers to >97%. This highly reversible postactivation behavior indicates that the chemically fused nanoparticle-nanofiber interface effectively stabilizes the initial SEI layer, limiting further irreversible electrolyte consumption during prolonged cycling. Although the initial Coulombic efficiency (ICE) is limited by intrinsic Li_2_O and SEI formation characteristic of conversion anodes,^31^ the focus of this study lies in the structural retention following this activation. The CV curves (Figure 7b) reveal a highly reversible alloying–dealloying process within the 0.1–1.0 V range.

The C-rate performance is shown in Figure 7c. PCNF-L demonstrated superior rate capability, recovering capacity even after cycling at 1000 mA g^–1^. The pure CNF experienced a ∼33.3% reduction in capacity as current increased, whereas PCNF–H and PCNF-L reduced by 66.7% and 57.1%, respectively. Notably, at 1000 mA g^–1^, PCNF–H reached the same performance as pure CNF, suggesting considerable resistance at high currents. The divergence in high-rate performance between PCNF–H and PCNF-L highlights a fundamental trade-off between active mass loading and transport kinetics. In PCNF-L, the higher loading of PbO (66.1%) increases the volume fraction of the less-conductive active material relative to the carbon percolation network. At high current densities (e.g., 1000 mA g^–1^), the denser packing of nanoparticles likely extends the solid-state lithium diffusion length and may create localized bottlenecks in electron transport, leading to increased overpotentials. Conversely, PCNF–H (53.2% loading) maintains a higher proportion of continuous carbon backbone (evident in its bimodal fiber distribution), providing more stable electronic conductivity and facilitating faster ion-transfer kinetics. Thus, while PCNF-L provides higher absolute capacity at low rates, PCNF–H demonstrates better transport retention under more demanding kinetic conditions.

EIS analysis (Figure 7e) further elucidated these results. The high-frequency region corresponds to lithium-ion diffusion resistance within the bulk electrode (*R*_b_) and the resistance through the solid–electrolyte interphase (*R*_SEI_), transitioning to charge-transfer resistance (*R*_ct_) in the midfrequency domain.^16,32^ The bulk resistances of PCNF–H, PCNF-L, and CNF were 13.6, 10.7, and 13.2 Ω, respectively, showing minor variation. However, the charge-transfer resistance (*R*_ct_) of the CNF sample (46.8 Ω) was lower than that of PCNF–H (83.2 Ω) and PCNF-L (62.4 Ω), which explains the faster performance degradation of PCNF electrodes during C-rate cycling compared with pure CNF. It is important to contextualize this comparison: pure CNF lacks the active metal oxide and its *R*_ct_ reflects kinetically rapid surface capacitive and intercalation reactions, albeit with lower specific capacity. In contrast, the PCNF samples undergo more complex multiphase conversion and alloying reactions (PbO → Pb + Li_2_O → LixPb), naturally resulting in a higher intrinsic *R*_ct_. The role of the interface in the CMD process is reflected in the observation that, even with a 66.1% loading of less conductive PbO, PCNF-L maintains an *R*_ct_ of 62.4 Ω, indicating that the hierarchical network sustains viable charge-transfer pathways. The relatively lower *R*_ct_ of PCNF-L compared to PCNF–H suggests a more effective incorporation and interfacial compatibility of PbO particles. This provides compelling evidence of enhanced charge kinetics facilitated by intimate heterointerfaces, which are superior to the weak point-contacts formed by simple physical adhesion.

Long-term cycling (Figure 7d) showed that PCNF-L maintained a high capacity of 466.8 mAh g^–1^ after 250 cycles, nearly double that of pure CNF (235.3 mAh g^–1^). Based on the mass loading, this specific capacity of 466.8 mAh g^–1^ corresponds to an operational areal capacity of approximately 0.9 to 1.4 mAh cm^–2^. PCNF–H, despite having lower loading, showed slightly higher retention (79.3% vs 72.8%). The degradation mechanism can be explained by a trade-off between mass loading and structural stress buffering. In PCNF-L, the high active material loading (66.1%) introduces a larger volume of PbO, which exerts significant chemo-mechanical stress on the CNF matrix during repeated lithiation expansion. This stress likely exceeds the local buffering capacity of the carbon scaffold in some regions, leading to microcracks and the eventual electrical isolation of some active particles (dead mass), which contributes to the capacity fade. Conversely, PCNF–H has a lower PbO loading (53.2%), meaning a higher ratio of carbon matrix to active material. This provides a more robust strain-buffering reservoir to accommodate the volumetric changes. Furthermore, the interpenetrating network of thin and thick fibers in PCNF–H acts as a redundant conductive pathway, bridging electrical contact even if localized fractures occur.^33–35^ Thus, while PCNF-L offers superior capacity through high loading, PCNF–H demonstrates that a stronger carbon backbone enhances retention.

To contextualize the structural advantages of the CMD architecture, the performance of PCNF-L was benchmarked against state-of-the-art Pb-based and conversion-type anodes (Table 1). While most conventional nanostructured Pb/C composites (e.g., core–shell or spray-pyrolyzed particles) exhibit severe capacity fading within 100 cycles due to particle agglomeration and isolation, the CMD-engineered PCNF-L maintains robust cycling over 250 cycles. More importantly, conventional vapor- or solution-based methods typically struggle to exceed a 50 wt % active material loading without structural failure. In contrast, the CMD process successfully embeds a high active mass loading of 66.1% (approximately 4–6 mg per electrode). This translates to a highly competitive areal capacity, demonstrating that the chemically fused PbO–CNF architecture does not sacrifice absolute energy metrics to achieve structural stability. This benchmarking highlights the efficacy of the liquid-phase fusion approach in producing practically viable, high-loading nanocomposites.

**Table 1:** Comparison of Mass Loading, Areal Loading, and Electrochemical Performance between the CMD-Engineered PCNF Anode and State-of-the-Art Pb-Based Nanocomposites

material system	synthesis method	mass loading (wt %)	areal loading (mg/cm^2^)	specific capacity (mAh/g)	cycle life	process time	yield
PBO–CNF (CMD)^this work^	dual-electrospinning	66.1%	∼2.0–3.0	466.8 (@200 mA/g)	250	<6 h	high
Pb/C microspheres^16^	hydrothermal	∼50%	∼2.6	852 (@500 mA/g)	850	>24 h	moderate
PbO/graphene Oxide^21^	coating/solvothermal	not reported	1.2–1.3	420 (@165 mA/g)	200	∼ 12 h	moderate
PbO/Cu–C matrix^36^	solvothermal	not reported	not reported	420 (@165 mA/g)	9500	>12 h	low
PbO nanofibers^37^	conventional electrospinning	not reported	not reported	372 (@100 mA/g)	50	∼10 h	moderate
PbO-carbon composite^38^	pyrolysis/carbonization	∼60%	∼2.0	∼480 (@100 mA/g)	200	>12 h	moderate
Pb-based nanocomposite^39^	melt-spinning/alloying	not reported	not reported	550 (@100 mA/g)	300	>12 h	moderate

Postcycling SEM characterization (Figure 8) visually validated the structural resilience and elucidated the failure modes of the different architectures. After 250 cycles, the average fiber diameters of PCNF–H, PCNF-L, and pure CNF increased by 126%, 66%, and 29%, respectively (Figure 8a,c,e). For PCNF–H (Figure 8a), the surface appears roughened with few visible particles, suggesting that the PbO particles—which were initially embedded due to rapid processing—were pulverized or electrically isolated within the matrix due to uneven volumetric expansion. This “dead mass” formation aligns with its lower capacity. In evaluating the structural resilience against the theoretical volume expansion of PbO, the postcycled PCNF-L fibers (Figure 8c) indicate that active particles remain structurally associated with the contiguous carbon fiber network. While capacity fade still occurs, the visual absence of severe catastrophic fracture or widespread agglomeration typically associated with unanchored Pb-based anodes supports the premise that the fused interface aids in accommodating volumetric strain. In contrast, PCNF-L (Figure 8c) reveals numerous PbO particles still robustly anchored to the CNF surface, coexisting with the solid–electrolyte interphase (SEI) layer. This confirms that the thermodynamically driven wetting created a robust interface capable of retaining active material even under significant strain. While the higher loading in PCNF-L imposes a greater mechanical burden, leading to a slightly faster decay rate than PCNF–H, its ability to maintain structural continuity without fragmentation proves the efficacy of the self-buffering CNF matrix, effectively mitigating the catastrophic failure typically associated with Pb-based anodes.^40^ The postrate capability images (Figure 8b,d,f) mirror these trends, showing that the hierarchical network remains intact even under high-current stress.

**Figure 8:**
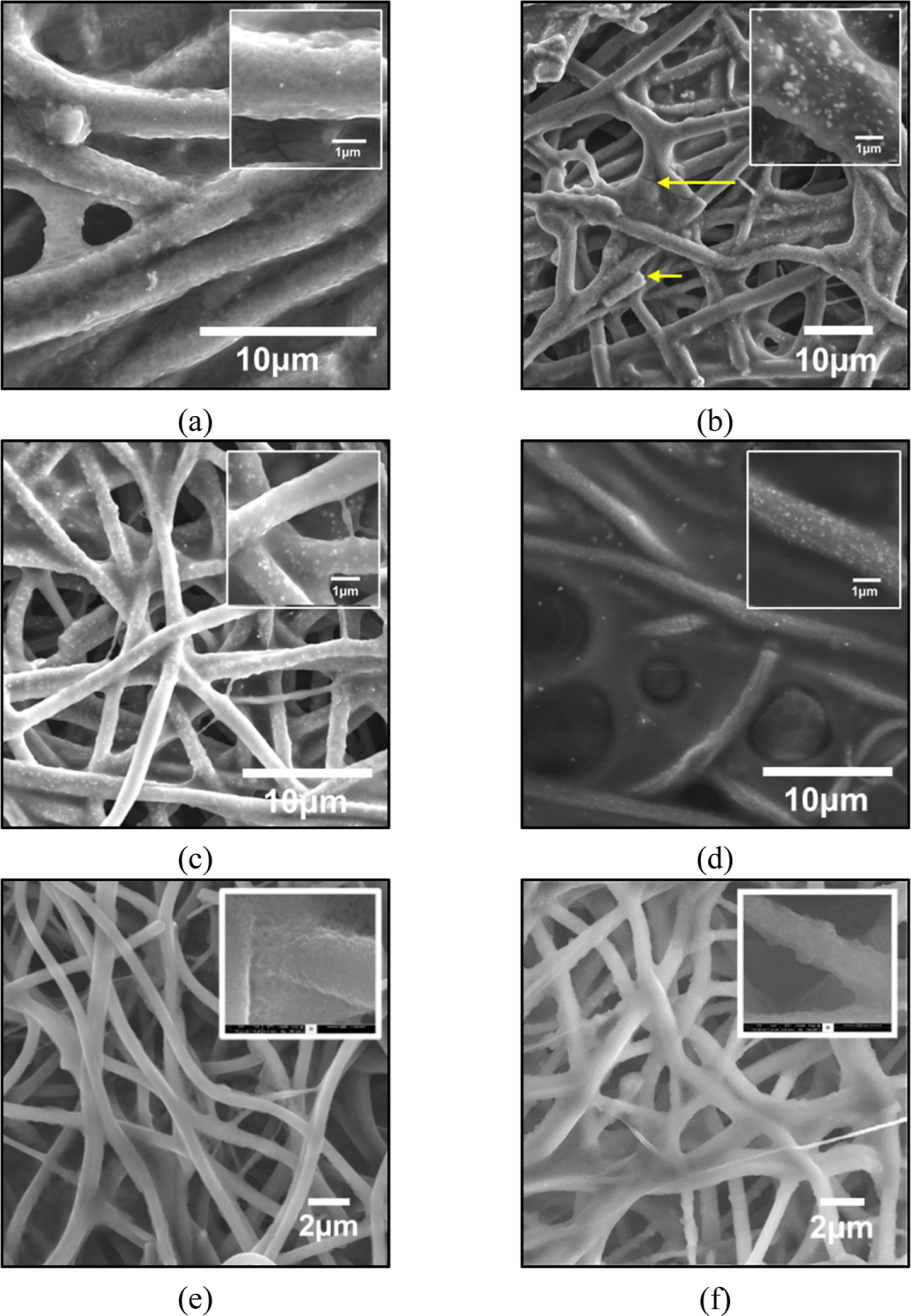
Post-mortem structural analysis. SEM images of (a) PCNF–H, (c) PCNF-L, and (e) pure CNF after 250 cycles; and (b) PCNF–H, (d) PCNF-L, and (f) pure CNF after rate-capability testing. Despite undergoing extreme volume expansion (up to 126%), the CMD-engineered architectures maintain a continuous, nonfragmented fiber network, demonstrating the efficacy of the strain-accommodating self-buffering matrix.

## 4 Conclusions

In summary, we have established CMD as a robust and scalable manufacturing method for the construction of high-yield nanoparticle–nanofiber architectures. By strategically exploiting the differential thermal behavior of dual polymer precursors, we demonstrated a TLPT mechanism that facilitates the chemical fusion of nanoparticles onto target scaffolds–a feat fundamentally unattainable through conventional line-of-sight vapor deposition or physical mixing. Using the high-expansion PbO/CNF system as a critical stress-test model, we proved that the CMD-engineered interface can successfully buffer extreme volumetric strain (∼233%) while maintaining exceptional charge-transfer kinetics. The resulting hierarchical, stress-dissipating architecture delivered nearly double the capacity of traditional carbonaceous anodes with superior long-term stability. Beyond the scope of lithium-ion storage, the fundamental principles of CMD established here provide a generalizable blueprint for the design of diverse hybrid systems, including next-generation catalysts, sensors, and multifunctional membranes, where robust interfacial connectivity is the primary bottleneck. We believe this liquid-phase integration strategy represents a significant step toward the efficient manufacturing of high-performance, structurally integrated nanomaterials.
